# A graphene-like BeS monolayer as a promising gas sensor material with strain and electric field induced tunable response: a first-principles study

**DOI:** 10.1039/d2ra08121k

**Published:** 2023-08-07

**Authors:** Akib Zaman, Rifat Shahriar, S. M. Takvir Hossain, Md Rajbanul Akhond, Homayra Tabassum Mumu, Ahmed Sharif

**Affiliations:** a Department of Materials and Metallurgical Engineering, Bangladesh University of Engineering & Technology Dhaka 1000 Bangladesh asharif@mme.buet.ac.bd (+88)-029665618 (+88)-01912802486; b Department of Electrical and Electronic Engineering, Bangladesh University of Engineering & Technology Dhaka 1000 Bangladesh

## Abstract

A comprehensive investigation of the gas sensing potential of BeS monolayer has been conducted using DFT calculations. Twelve common pollutant gases: NH_3_, NO_2_, NO, CO, CO_2_, CH_4_, H_2_, O_2_, N_2_, H_2_S, H_2_O and SO_2_, have been studied. Our analysis reveals defect states in the band structure near the Fermi level and strong hybridization between gas molecule orbitals and the BeS monolayer. We observe higher adsorption energies for NH_3_ and CO compared to other popular gas sensing materials. The optical properties of CO_2_ and NO_2_ adsorbed on the BeS monolayer show increased reflectivity and absorption coefficient in the UV and far infrared region. Tensile strain has minimal impact on adsorption energy, while biaxial compressive strains enhance the gas sensing capability of the BeS monolayer. The application of an electric field offers control over gas adsorption and desorption. We propose the BeS monolayer as a promising candidate for future gas molecule sensing applications due to its high adsorption energy, rapid recovery time, and distinct optical properties.

## Abstract 2

Precise manipulation of gas adsorption and desorption in the BeS monolayer can be carried out by means of an electric field, such as a gate voltage in a 2D material transistor.

## Graphical abstract

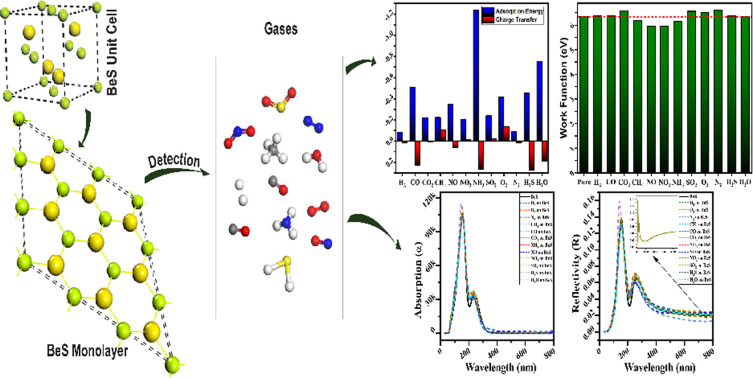

## 1 Introduction

Natural toxins can cause serious wellbeing issues like eye irritation, asthma, respiratory congestion, cardiac problems, cancer and diminish the working of the pneumonic framework and are moreover perilous for other living life forms and, indeed, for the entire environment.^1^ Thus it has been an imperative assignment for researchers to discover successful strategies and methods to identify and expel such toxins from the air.^2^ Gas sensors that can identify various types of gaseous pollutants are of remarkable significance. Researchers have now developed different types of gas sensors such as oxide semiconductor, organic type semiconductor, field effect style, surface acoustic wave nature and so on.^3–6^ Extensive research has been performed to theoretically predict and synthesize monolayer forms of bulk 3D materials.^7–9^ 2D materials have been used in various applications such as topological insulators,^10–14^ spintronic devices,^15,16^ memory devices,^17^ gas sensor devices^9,11,18–31^*etc.* 2D materials demonstrate several exotic properties, such as quantum confinement,^32^ novel topological phases,^33^ and electron super collimation.^34^ Generally, MOS gas sensors have been widely used in industry. However, in small molecule sensing, the performance of MOS gas sensors is rather poor. Hence, plenty of novel 2D materials have been considered for gas sensing applications.

Recently, many III–V bi-elemental monolayers have been considered for gas sensing materials such as InN, h-BAs, h-BN.^35–37^ The InN monolayer has been proven to be an excellent SO_2_ sensor. Group IV bi-elemental and mono-elemental materials have also generated significant interest among researchers. Recent work on Stanene showed that it can be used as an effective sensing material for NO and NO_2_ gases.^38,39^ Bi-elemental group IV materials such as SiC bilayer have been theoretically predicted to be useful as an optical sensor of NH_3_.^18^ However, group II–VI monolayers such as BeS, BeSe, MgO have received scant attention. Recently, it has been predicted theoretically that BeS monolayer has a large bandgap with excellent electronic and optical properties.^40^

In this theoretical work, the prospect of using a BeS monolayer as a gas sensor has been explored. We attempt to examine the adsorption of twelve common pollutant gases—H_2_, CO, NH_3_, CH_4_, CO_2_. SO_2_, NO, O_2_, N_2_, H_2_S, H_2_O and NO_2_ on BeS monolayer using first-principles approach based on density-functional theory (DFT). Adsorption energy values were calculated to determine the stability of adsorption. Density of states calculations were performed to elucidate the chemical interaction between gas molecules and the BeS monolayer. Optical properties and recovery time of gas adsorbed systems were determined to evaluate the sensitivity and reusability of the BeS monolayer as a gas sensing material. This study might provide useful guidelines for future experimental research on the BeS monolayer as a gas sensor for toxic molecule sensing.

## 2 Computational details

In this study, DFT calculations have been carried out using the DMol^3^ (ref. 41) and CASTEP (ref. 42) package of Materials Studio. CASTEP module has been used to calculate the phonon spectra of pristine monolayer to verify the dynamic stability of the planar structure. Electron density difference and optical properties have also been calculated using CASTEP. Ultrasoft pseudopotentials have been used for wave function expansion. *K*-Point sampling was 9 × 9 × 1 for phonon dispersion calculation, 16 × 16 × 1 for optical properties calculation, and 5 × 5 × 1 for electron density difference calculation.

Geometry optimization, band structure, density of states, and Mulliken charge analysis of 3 × 3 supercell of pristine and gas adsorbed BeS monolayer have been done with Dmol^3^ module. Monkhorst Pack grid of 10 × 10 × 1 and 16 × 16 × 1 was used for geometry optimization and electronic structure calculation, respectively. In geometry optimization, each structure was relaxed until energy and force were converged to 1.0 × 10^−7^ Ha and 1.0 × 10^−4^ Ha per Angstrom, respectively. Fermi level smearing was taken as 0.005 Ha. Double Numerical Polarized (DNP) basis set was used with a global orbital cutoff of 5.0 Å.^11^ To minimize spurious interaction with periodic images, a large vacuum slab of 20 Å was used. Generalized Gradient Approximation with Perdew Burke Ernzerhof functional^43^ and DFT-D correction method proposed by Grimme^44,45^ has been used in all calculations. The gas molecules were placed on four different sites. Most preferred orientation of the gas molecules as found in the literature has been used as the initial geometry guess.^46,47^ Then the initial structures were relaxed until the convergence thresholds were met. The electronic and optical property calculation of the most stable structures were then subsequently calculated.

Adsorption energy of gas adsorbed systems were calculated using the following equation:^48^

1 *E*_ad_ = *E*_gas+BeS_ − *E*_BeS_ − *E*_gas_

Here, *E*_ad_, *E*_gas_, *E*_BeS+gas_, and *E*_BeS_ represents adsorption energy, the energy of gas molecule, gas adsorbed BeS monolayer, and BeS monolayer, respectively. A negative value of *E*_ad_ denotes strong interaction and a stable adsorption structure. The larger the absolute value of *E*_ad_, the stronger the interaction.

The charge transfer, Δ*ρ* between the gas molecules and the BeS monolayer were calculated using the following equation:

2 Δ*ρ* = *ρ*_BeS+gas_ − *ρ*_BeS_ − *ρ*_gas_

Here, *ρ*_BeS+gas_, *ρ*_gas_ and *ρ*_BeS_ represents the charge distribution of the gas adsorbed BeS monolayer, gas molecules and pristine BeS monolayer, respectively.

## 3 Result and discussion

### 3.1 Pristine monolayer

**Fig. 1:**
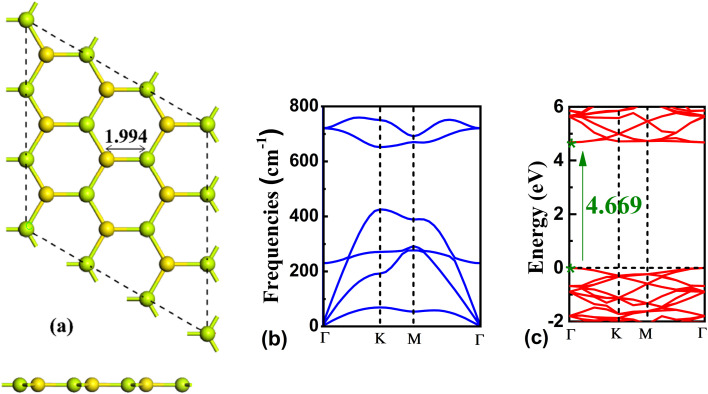
(a) The top and side view, (b) phonon spectra, and (c) band structure of the pristine BeS monolayer.

Before analyzing the properties of BeS analyte systems, the geometric and electronic structure of pristine BeS monolayer was obtained. BeS monolayer was found to be a hexagonal honeycomb monolayer with a bond length of 1.994 Å (Fig. 1(a)). The band gap of BeS monolayer at the GGA-PBE level of theory is 4.669 eV. The bandgap is in good agreement with previous study.^46^ PBE functional is known for underestimating the band gap of semiconductors. However, for sensing purposes, the change in the band gap is more important than the absolute value. Hence, GGA-PBE functional is used for the band structure calculation of the gas adsorbed systems. Generally, 2D planar structure shows dynamic instability and the structure with an imaginary phonon dispersion indicates there would be a transition to a relatively more stable buckled structure by atomic displacements along the vertical direction of the 2D plane.^49^ Thus, the dynamic stability of the planar BeS monolayer was verified by calculating the phonon spectra. From Fig. 1(c), it can be seen that no imaginary frequency is present in the phonon spectra. Thus, the BeS monolayer has a stable planar structure.

### 3.2 Adsorption configuration of gas molecules on BeS ML

**Fig. 2:**
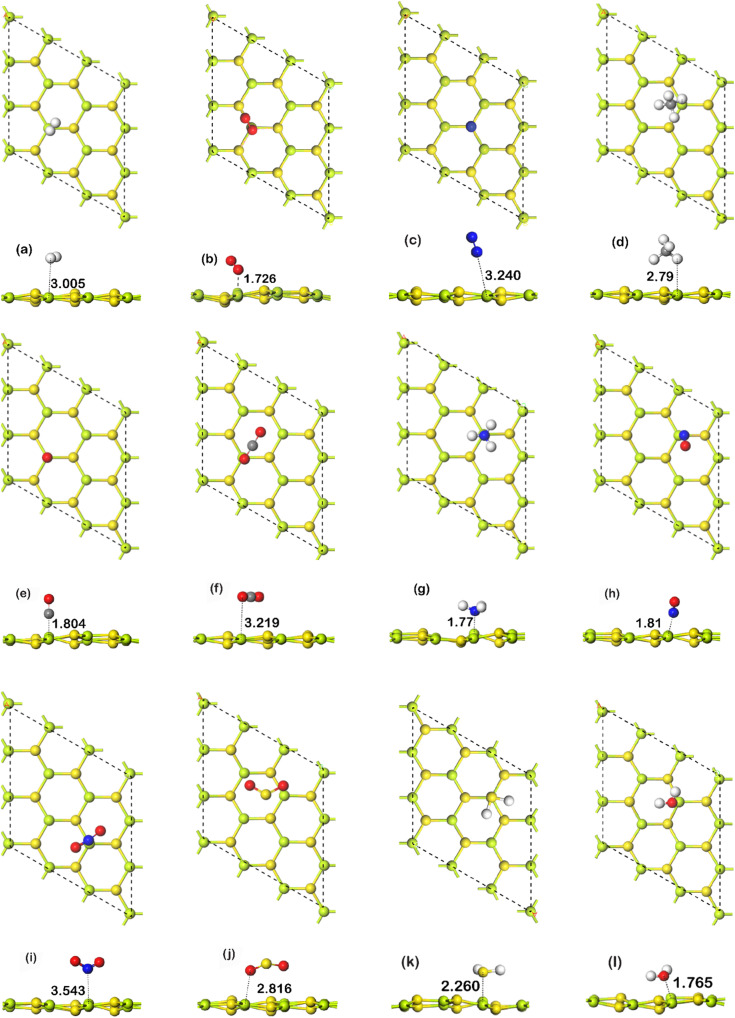
(Top/side view) the most energetically favorable adsorption configurations of the adsorbed molecules (a) H_2_, (b) O_2_, (c) N_2_, (d) CH_4_, (e) CO, (f) CO_2_, (g) NH_3_, (h) NO, (i) NO_2_ (j) SO_2_, (k) H_2_S, (l) H_2_O on the BeS monolayer surface (atom color code: blue, nitrogen; green, beryllium; yellow, sulfur; white, hydrogen; red, oxygen; grey, carbon).

For the study of gas adsorption, four sites were considered. The sites are: Be, S, hollow, and bridge. Here, hollow indicates the space between any hexagonal ring of the monolayer, and bridge indicates the bond between Be and S atom. In bridge configurations, the gas molecules were initially placed over the center of the bonds. Only the most stable site has been further considered for electronic and optical structure evaluation. In Fig. 2, all of the fully relaxed gas adsorbed structures are shown. In Table 1, adsorption energy, charge transfer, work function, adsorption distance, and recovery time have been summarized. In the table, *D* is defined as the shortest distance between the gas molecule(s) and the monolayer. *Q* is defined as the Mulliken charge transfer between the analyte and BeS. Here a positive sign indicates that the gas molecule acts as a donor to the substrate, whereas a negative sign indicates that the gas molecule acts as an acceptor to the substrate. Recovery time is the time required for a sensor to return to its base state. Two categories of adsorption could occur: physisorption and chemisorption. In chemisorption, chemical interaction occurs between gas molecule and substrate material. When chemisorption occurs, adsorption energy and charge transfer are high, and strong charge density would be observed between the substrate monolayer and gas molecule. In physisorption, the adsorption energy is lower, the gas molecule is bound to the substrate with weak van der Waals force, and there is no strong charge density between the substrate monolayer and gas molecule.

**Table 1:** The adsorption value of BeS monolayer for H_2_, CO, CO_2_, CH_4_, NO, NO_2_, NH_3_, SO_2_, H_2_S, H_2_O, N_2_ and O_2_. *E*_ad_ (eV): the adsorption energy. *Q* (e): the charge transfer between gas molecule and BeS monolayer. *D* (Å): the shortest distance of the atom in the molecule to the BeS surface: recovery time in second: work function (eV)

Gas	*E*_ad_ (eV)	*Q* (e)	*D* (Å)	Recovery time (s)	Work function (eV)
None	—	—	—	—	6.34
H_2_	−0.08	0.015	3.005	2.20 × 10^−11^	6.37
CO	−0.51	0.227	1.804	3.56 × 10^−4^	6.37
CO_2_	−0.22	0.005	3.219	4.89 × 10^−9^	6.585
CH_4_	−0.225	−0.103	2.791	5.93 × 10^−9^	6.18
NO	−0.35	0.063	1.81	7.39 × 10^−7^	5.96
NO_2_	−0.205	−0.01	3.543	2.74 × 10^−9^	5.96
NH_3_	−1.24	0.268	1.777	6.20 × 10^8^	6.15
SO_2_	−0.24	−0.022	2.816	1.06 × 10^−8^	6.56
H_2_S	−0.452	0.274	2.26	3.79 × 10^−5^	6.37
H_2_O	−0.755	0.186	1.765	4.57	6.34
N_2_	−0.089	0.0133	3.24	3.107 × 10^−11^	6.61
O_2_	−0.415	−0.14	1.726	9.09 × 10^−6^	6.503

In the case of H_2_ adsorption on BeS, the most favorable adsorption site is somewhere between the bridge site and Be site. In both cases, during geometry optimization, the gas molecule moved to the site between the center of the bond and the Be atom. The observed adsorption distance was 3.005 Å, and the charge transfer 0.015 |*e*|. The most energetically favorable adsorption configuration has adsorption energy of −0.08 eV. This value is not very high. The values of adsorption energy, charge transfer, and adsorption distance indicate that the adsorption is physisorption nature. When CO is adsorbed on BeS, the most stable adsorption site is Be site. The bond calculator tool of Materials Studio indicates that a chemical bond is formed between the Be atom and the C atom, which is shown in Fig. 2(b). The distance between the C atom and the Be is 1.804 Å. Additionally, 0.227 |*e*| charge gets transferred from the gas molecule to the monolayer. The adsorption energy of −0.51 eV is also fairly high. These results indicate that the CO atom was chemisorbed on the substrate. Contrary to CO, when CO_2_ was adsorbed on the BeS monolayer, the adsorption energy was found to be only −0.22 eV. The calculated adsorption distance was reasonably large—3.219 Å. The charge transfer from CO_2_ to BeS monolayer was only 0.005 |*e*|. The results mean that CO_2_ was weakly physiosorbed on the BeS monolayer. In the case of CH_4_, the adsorption occurs on the hollow site. The adsorption distance is 2.791 Å. A Mulliken charge transfer of 0.103 |*e*| from the BeS monolayer to the gas molecule is observed. So, CH_4_ acted as an acceptor. The adsorption energy is −0.225 eV which is not very high. All these results demonstrate that the adsorption of CH_4_ on the BeS monolayer is of a physisorption nature. For NO on BeS, the preferred adsorption site is Be. The shortest distance from the gas molecule to the monolayer is 1.81 Å. The Mulliken charge transfer is 0.063 |*e*| and the adsorption energy of the most energetically favorable configuration is −0.35 eV. The adsorption energy is moderate, and the adsorption distance was low. The results suggest that the adsorption of NO on BeS was moderately strong. On the other hand, when NO_2_ was adsorbed on BeS, the adsorption distance was pretty high—3.543 Å. The charge transfer was negligible (−0.01 |*e*|). The adsorption energy, however, was found to be −0.205 eV, which indicates a stable configuration. Hence, the adsorption of NO_2_ is weak, however, chemical interaction between the monolayer and the molecule might have occurred. For NH_3_ on BeS, NH_3_ was adsorbed on the Be atom of BeS. Here, the adsorption distance between the N atom and the Be atom was the shortest distance, which is 1.777 Å. Mulliken charge transfer from the NH_3_ molecule to the BeS monolayer was 0.268 |*e*|. The adsorption energy was −1.24 eV, which is also very high. The high adsorption energy, high charge transfer, and short adsorption distance indicate the adsorption of NH_3_ on BeS is chemisorption nature. The results demonstrate that the BeS monolayer can be an excellent candidate for NH_3_ sensing. When SO_2_ was adsorbed on BeS, the S atom was directly over the hollow site, and the O atoms were close to the Be sites. The interaction between the gas molecule and the monolayer was weak, which is indicated by the low charge transfer of −0.022 |*e*|. The distance between the molecule and the monolayer was 2.816 Å which is fairly large. Although the adsorption energy of −0.24 eV is moderate, the small charge transfer and large adsorption distance indicate physisorption nature. For H_2_S, the most stable adsorption site is the Be site where we found the minimum adsorption energy which is −0.452 eV. The charge transfer and adsorption distance are 0.274 |*e*| and 2.26 Å respectively. The high value of adsorption energy suggests that H_2_S is chemically absorbed on the monolayer. However, the H_2_O shows higher adsorption energy of −0.755 eV at S site. The charge transfer of 0.186 |*e*| and adsorption distance of 1.765 Å further suggests the chemisorption of H_2_O. N_2_ being adsorbed at bridge site is weakly physiosorbed because the adsorption energy (−0.089 eV) is very low, and the distance of adsorption (3.24 Å) is high. The charge transfer (0.0133 |*e*|) is also not very high. The O_2_ gas shows an adsorption energy of −0.415 eV. It was most stable at Be site at 1.726 Å from the substrate. The negative charge transfer of −0.14 |*e*| indicates that O_2_ is a moderate acceptor of electrons compared to the monolayer. Though note that most of the gases adsorbed on the monolayer has an adsorption energy lower than −0.5 eV. This means that those gas molecules could readily be released from BeS monolayer. This indicates excellent reusability of BeS monolayer. Considering adsorption energy and charge transfer, the monolayer is selective to NH_3_ and CO where the adsorption of NH_3_ is significantly stronger than CO. Though H_2_O was chemisorbed to the substrate, considering the other properties the selectivity of H_2_O was not regarded.

**Fig. 3:**
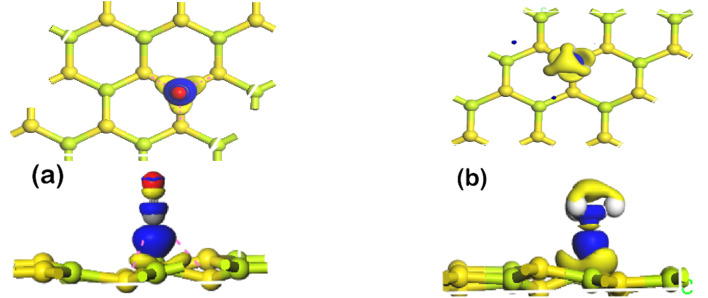
Charge density difference plots for (a) CO, (b) NH_3_, interacting with BeS monolayer. The purple (yellow) distribution corresponds to charge accumulation (depletion). The isosurface is taken as 0.02 e Å^−3^ for (a) & (b).

To further elucidate the nature of adsorption of CO and NH_3_ on BeS monolayer, electron density difference has been calculated. The electron density difference plots of CO and NH_3_ adsorbed monolayers are shown in Fig. 3(a and b). In electron density difference illustration, blue color denotes electron accumulation, and yellow color denotes electron depletion. In the electron density difference calculation, charge piling up between the gas molecule and BeS monolayer has been observed. High charge transfer is an indication of chemisorption. Therefore, from the results of adsorption energy, adsorption distance, charge transfer, and electron density difference, a conclusion can be drawn that NH_3_ and CO gas molecules have chemisorbed on BeS monolayer. In Table 2, a comparative study of adsorption energy and charge transfer between CO/NH_3_ adsorbed BeS monolayer, and some other monolayers has been presented. BeS monolayer has significantly higher adsorption energy and charge transfer during NH_3_ and CO adsorption than popular 2D materials, such as stanene, WSe_2_, SiC, BAs, GaTe or InN. This might be an indication that BeS monolayer could be a better sensing material than the other monolayers for detecting NH_3_ and CO.

**Table 2:** Adsorption energy, *E*_ad_ (eV) and charge transfer, *Q*(|*e*|) of CO/NH_3_ adsorbed BeS monolayer and some other monolayers

2D material	Adsorption energy, *E*_ad_ (eV)		Charge transfer, *Q*(|*e*|)	
	CO	NH_3_	CO	NH_3_
BeS (this work)	−0.51	−1.24	0.227	0.268
Stanene^a^	−0.121	−0.438	0.101	−0.1035
SiC^b^	−0.12	−0.83	0.08	0.316
WSe_2_^c^	−0.0092	−0.042	0.0092	0.0187
GaTe^d^	−0.125	−0.289	−0.01	−0.01
InN^e^	−0.223	−0.859	0.033	0.103
BAs^f^	−0.27	−0.34	−0.024	0.007

a Ref. 39.

b Ref. 18.

c Ref. 28.

d Ref. 31.

e Ref. 11.

f Ref. 36.

### 3.3 Electronic properties of gas adsorbed BeS ML

**Fig. 4:**
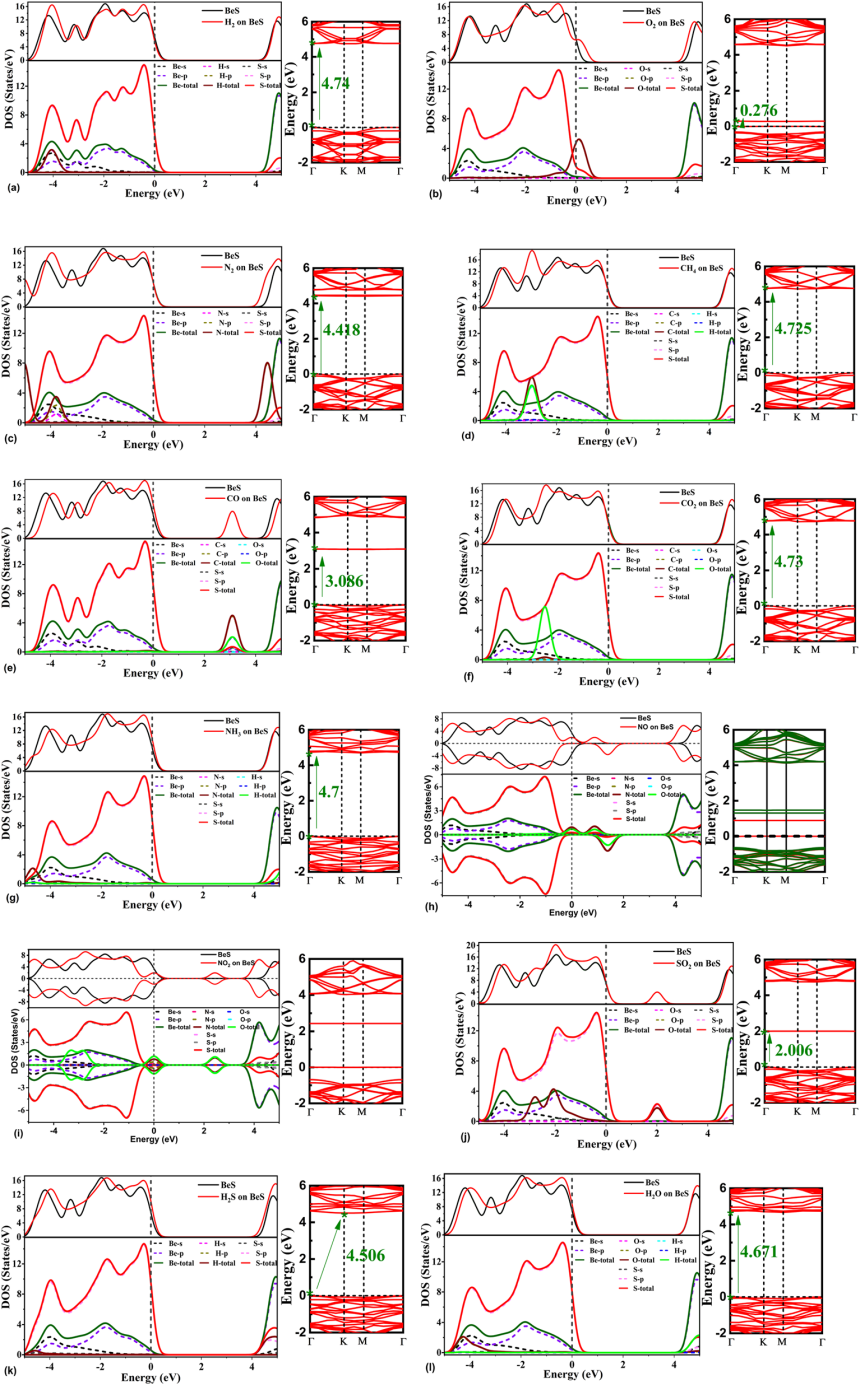
The total and partial densities of states and band structure for (a) H_2_, (b) O_2_, (c) N_2_, (d) CH_4_, (e) CO, (f) CO_2_, (g) NH_3_, (h) NO, (i) NO_2_ (j) SO_2_, (k) H_2_S, (l) H_2_O on the BeS monolayer surface based on the most stable configuration.

For getting a deeper understanding of the effects of gas adsorption on the electronic properties of BeS monolayer, the total and partial electronic densities of states and band structures were also analyzed. The density of states and band structures have been shown in Fig. 4(a–l). The adsorption of H_2_ does not affect the density of states around the Fermi level. However, s orbitals of H atoms do hybridize with s and p orbitals of Be and S atoms at −4 eV, which alters the total density of states at −4 eV. The bandgap reduces significantly for O_2_ adsorption. This reduction is due to the defect states generated by the p orbitals coming from the O_2_ molecule. N_2_ also didn't perturb the density of states near the Fermi level, so the bandgap didn't vary much. However, hybridization occurs near −3.8 eV among p orbitals of N and S, and s orbital of Be. In CO adsorption, significant decrease of band gap was observed. The reduction in band gap is due to the defect states arising at the 2 to 4 eV range. At this range, orbital contribution from Be or S atoms were found to be negligible. But p orbitals of C and O atoms give rise the aforementioned defect states. When CO_2_ is adsorbed on BeS monolayer, defect states are observed at around −2.5 eV. The defect states are caused by p orbitals of O atoms. At this range, p orbitals of O atoms show strong hybridization with p orbitals of both Be and S atoms. When CH_4_ is adsorbed on the monolayer, p orbitals of C atom and s orbitals of H atoms hybridize with the p orbitals of S atoms. Hence, a defect state arises at the −2 eV to −4 eV range. It was observed that the density of states around the Fermi level reduced when NO_2_ and NO were adsorbed. These two molecules significantly reduced the bandgap. From band structure and density of states plots in Fig. 4(e and f), it can be noticed that NO and NO_2_ both introduce defect states near Fermi level, which mainly stems from the p orbitals of N and O atoms. When NO molecule is adsorbed on BeS, there is a slight asymmetry in spin-resolved density of states and a split of up spin and down spin. According to Mulliken spin analysis, the total spin is 0.997 a.u. When NH_3_ is adsorbed, there is a small increase in the density of states near the top of the valence band. No significant contribution of N or H atom is found near the Fermi level, which explains why bandgap change is minimum when NH_3_ is adsorbed. In the case of SO_2_ adsorption, there is a noticeable change in DOS in the −3 to −1 eV region. From the band structure, a defect state in this region can be observed, which leads to the conclusion that the defect state arises from SO_2_. For H_2_S, the density of states at the valence band maxima slight increased after adsorption which can be due to the p orbital of S. The contribution of H and S is less near the Fermi level for which the bandgap didn't alter much. However, the bandgap is now indirect type after the adsorption of H_2_S. The effect of H_2_O in terms of changing density of states near Fermi level or bandgap is negligible. However, from −3.5 eV to −4.5 eV p orbitals of O, S hybridize with s orbital of Be.

### 3.4 Optical properties of gas adsorbed BeS monolayer

Optical gas sensors have gained popularity recently with advances in plasmonics and nano photonics.^39^ 2D materials-based optical gas sensors are also on the horizon. Hence it is important to investigate whether BeS monolayer can be effective as a gas sensor material with discriminate optical properties to disparate gases. The absorption coefficient and reflectivity have been calculated for all gas adsorbed systems.

Firstly, work function of BeS monolayer was calculated using formula (3):^50^

3 *Φ* = *V*_∞_ − *E*_F,_

where, *V*∞ and *E*_F_ are the electrostatic potential and the fermi energy level, respectively. Basically, the work function describes the minimum energy needed to dislodge an electron from the surface of BeS monolayer. The calculated work function of BeS monolayer was 6.34 eV. This is higher than most monolayers, including Graphene, h-BN, h-BAs, and GaAs.^36,51–54^ Upon gas adsorption, the work function did not vary much in the cases of CO and H_2_. NO, NO_2_, NH_3_, and CH_4_ adsorption reduced the work function, while the greatest reduction occurred when NO or NO_2_ was adsorbed. SO_2_ and CO_2_ increased the work function.

**Fig. 5:**
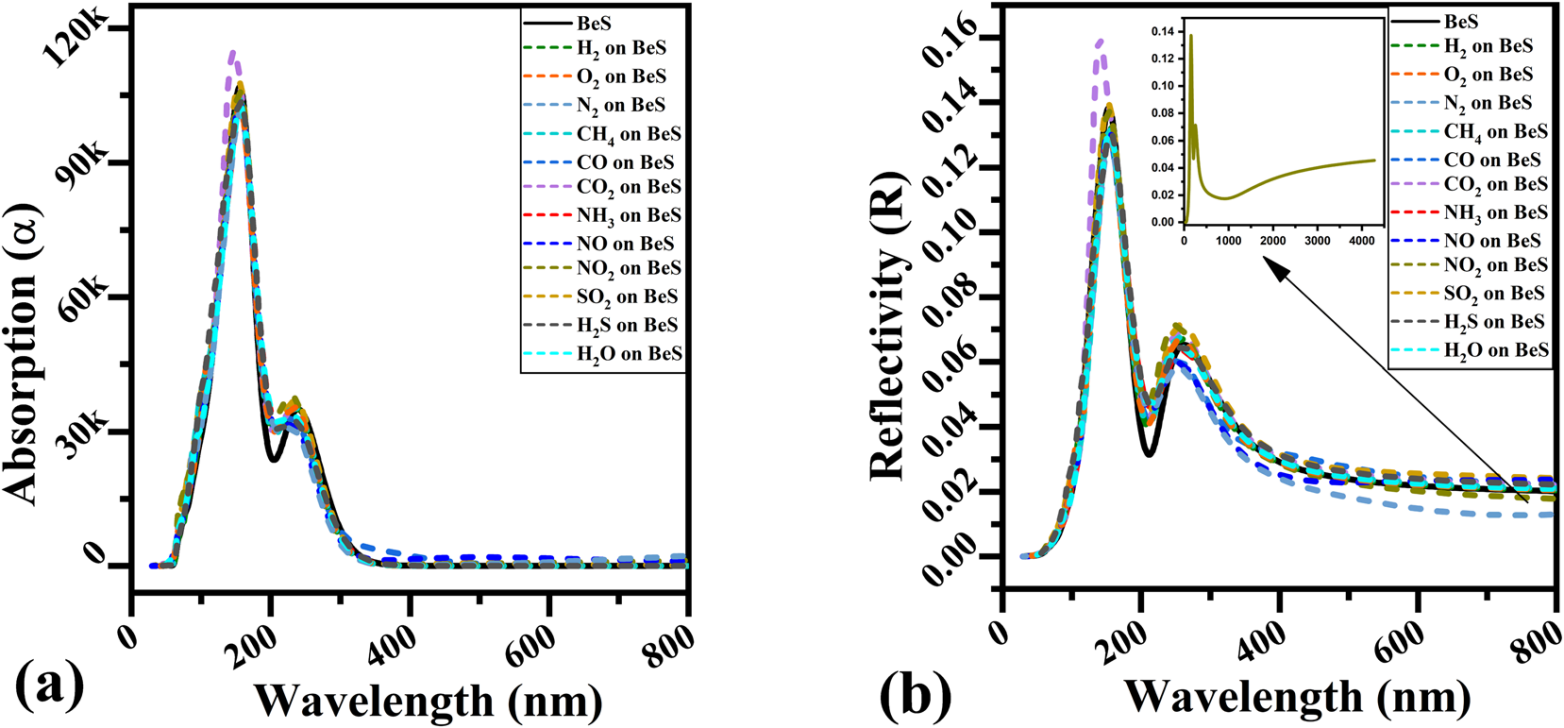
The calculated (a) absorption and (b) reflectivity of different gas molecules absorbing on BeS systems for the polarization vector perpendicular to the surface. And the inset of (b) is the reflectivity of NO_2_ on BeS surface within the wavelength range 0–4000 nm.

Secondly, adsorption co-efficient and reflectivity were calculated and shown in Fig. 5(a and b). Due to the low thickness of the material, reflectivity is more important than absorption co-efficient. Two major adsorption peaks are located in the ultra violet region which arise at around 150 nm and 230 nm. In the 100 to 200 nm region, CO_2_ adsorbed BeS monolayer has a 11% higher reflectivity and 10% higher absorption co-efficient. As can be seen in Fig. 5(a), in the 200 to 280 nm region, NH_3_ and NO adsorbed system does not have visible through that other gas analyte systems have. For reflectivity, two major peaks were also found. In the far-infrared range, NO_2_ adsorbed system has higher reflectivity which is shown in the inset of Fig. 5(b) which is similar to NO_2_ adsorbed InN monolayer.^11^ By tuning the monolayer to the appropriate frequency, an optical gas sensor can be synthesized by using this discriminate optical property of gas adsorbed BeS monolayer systems.

### 3.5 Recovery time

A crucial parameter of any gas sensor is the recovery time. For application purposes, a gas sensor has to be frequently reusable. Hence, the material used in the sensor should demonstrate the property of a low recovery time. Recovery time has been calculated using the following formula:^55^4
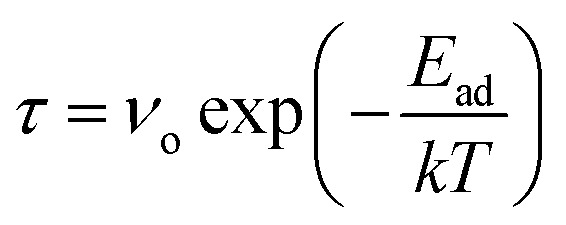
where, *ν*_o_ is the attempt frequency and *E*_ad_ is the adsorption energy. The attempt frequency has been assumed to be 10^−12^ s^−1^ for all gas molecules in this study. Recovery time has been summarized in Table 1. The recovery time of BeS monolayer was found to be low in the case of all the molecules except NH_3_ in this research. So, when NH_3_ will be adsorbed in this system, the adsorbed molecules will take a longer time for desorption. The long desorption time can be shortened by applying electric field and strain as these methods have been proven to effective in reducing the adsorption energy in some cases.^27,39^

### 3.6 Conductivity

Chemiresistive sensors have been used for gas molecule sensing for a long time. Ambient gas molecules usually change the resistivity of a material which can be used as a sensing parameter to detect the presence of toxic gases. The conductivity (*σ*) of a material can be expressed as5
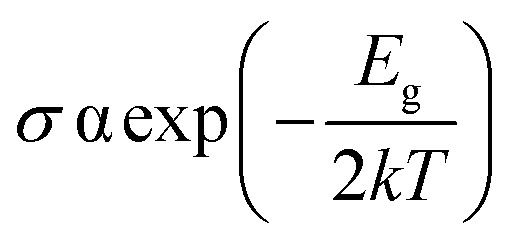


Using the above formula, the change in conductivity can be defined as6
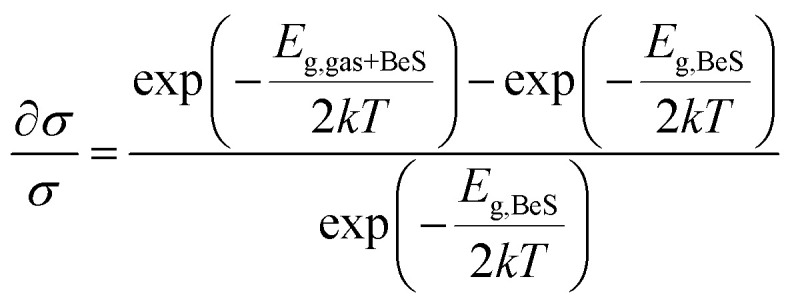
Here, *k* is the Boltzmann constant and *T* is ambient temperature (300 K for this study). *E*_g,gas+BeS_ and *E*_g,BeS_ are the bandgap of gas adsorbed BeS monolayer and pristine BeS monolayer. The equation describes the relationship between band gap and conductivity. The larger the change in band gap, the larger the change in conductivity. From Fig. 4, it is clearly seen that the largest change in band gap occurs when NO and NO_2_ are adsorbed on the BeS monolayer. Additionally, a moderate change of conductivity is observed in the CO absorbed BeS monolayer too. Resistive gas sensors observe the change in resistivity as a sensing mechanism. By monitoring the change of resistivity, small gas molecules could be easily detected. For a resistive gas sensor detecting NO, NO_2_, or CO gas molecule, BeS monolayer could therefore be an ideal choice.

### 3.7 Improvement of sensing response with strain

**Fig. 6:**
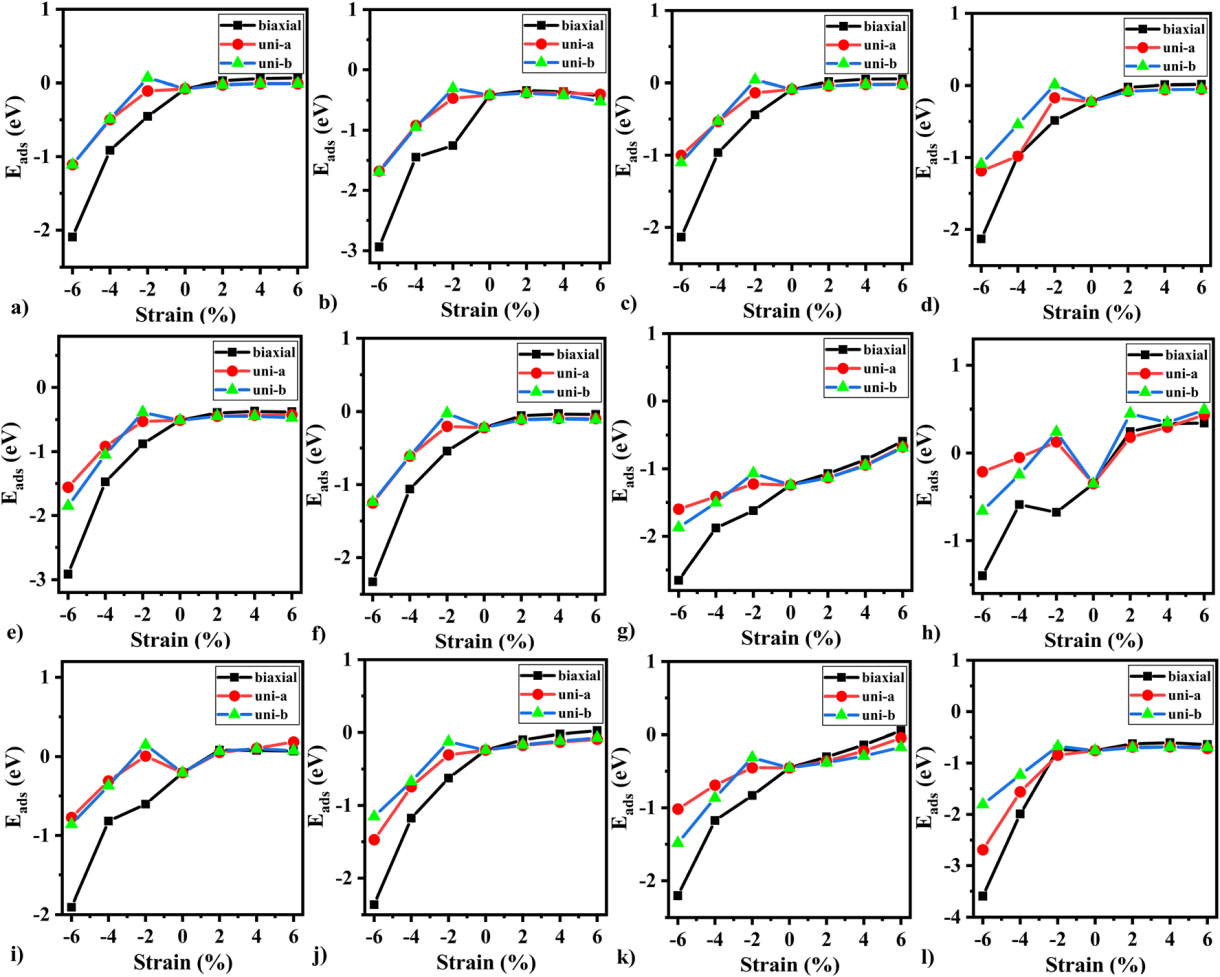
The calculated adsorption energy as a function of applied strain for (a) H_2_, (b) O_2_, (c) N_2_, (d) CH_4_, (e) CO, (f) CO_2_, (g) NH_3_, (h) NO, (i) NO_2_ (j) SO_2_, (k) H_2_S, (l) H_2_O adsorbed at BeS monolayer.

We applied strain (biaxial and uniaxial) on the relaxed monolayers to improve the sensing response. The value of strain can be expressed as *ε* = (*a* − *a*_0_)/*a*_0_, where *a* is the lattice parameter of BeS monolayer. For biaxial strain, the two lattice parameters a and *b* are equal. In contrast, for uniaxial strain, lattice constant was held constant for one axis, and was varied for the other. As can be seen in Fig. 6, the trend of adsorption energy was similar for all gas molecules. Tensile strain did not improve the strength of adsorption. In fact, for NO (see Fig. 6(h)), the adsorption energy became a positive quantity which signifies that NO can be expected to desorb from BeS monolayer under a tensile strain greater than 2%. In contrast, compressive strain did improve the strength of adsorption for all of the gas molecules. In all cases, for a biaxial compressive strain of >2% causes a transition from physisorption to chemisorption. Uniaxial strain also improved the strength of adsorption. However, the response was better for biaxial strain. Hence, we can conclude that for BeS monolayer, biaxial compressive strain could be an effective method of improving the gas adsorption (and consequently the gas sensing) performance.

### 3.8 Improvement of sensing response with a vertically applied electric field

**Fig. 7:**
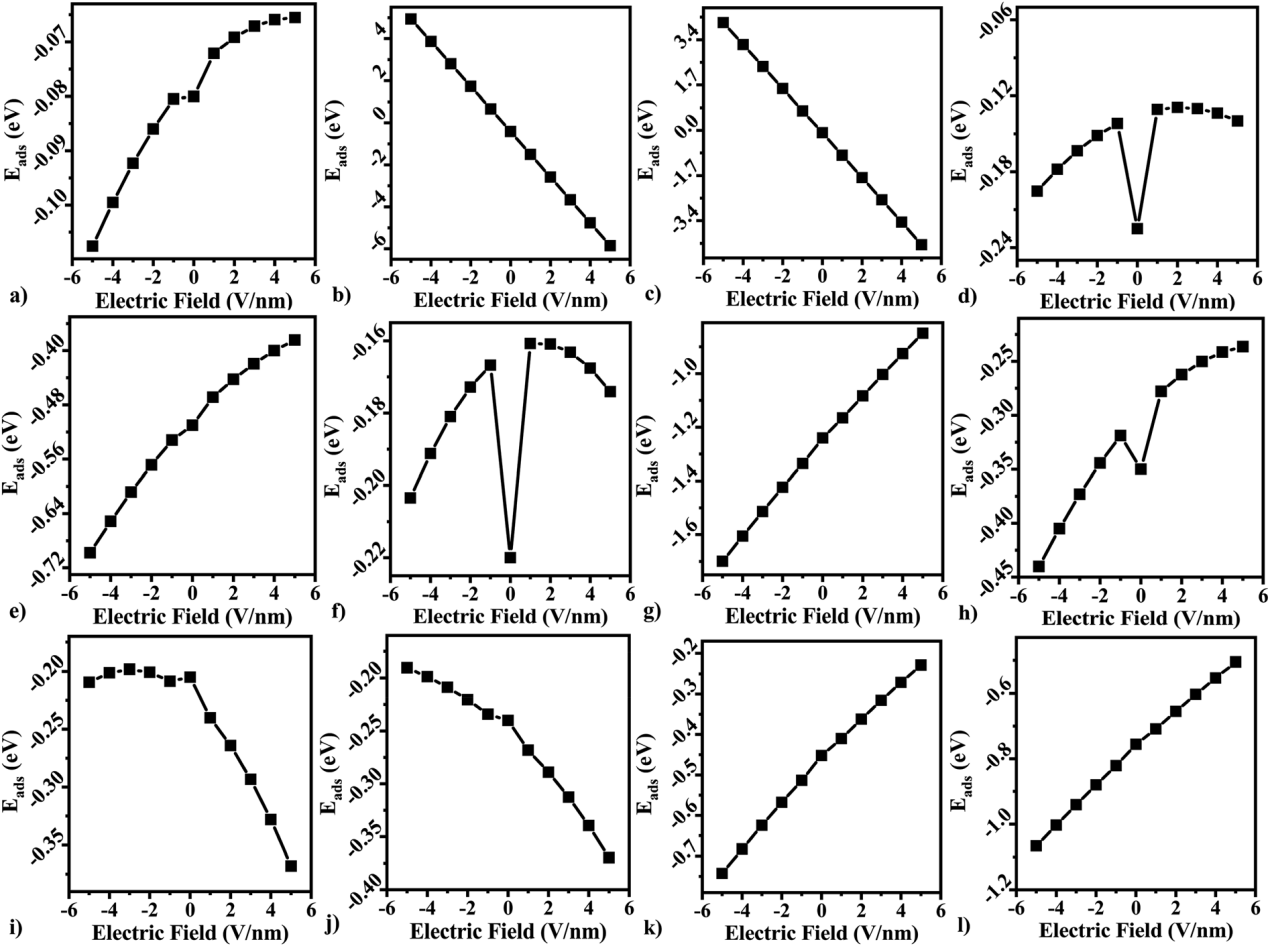
The calculated adsorption energy as a function of applied external electric fields (E-field) for (a) H_2_, (b) O_2_, (c) N_2_, (d) CH_4_, (e) CO, (f) CO_2_, (g) NH_3_, (h) NO, (i) NO_2_ (j) SO_2_, (k) H_2_S, (l) H_2_O adsorbed at BeS monolayer and the external E-field is perpendicular to the plane of BeS monolayer with its positive direction aligned upward to the coordinate axes.

In three terminal devices such as transistors, the vertical electric field on the material plays a crucial role. We modelled the effect of gate voltage by applying an electric field on the gas adsorbed and pristine free standing BeS monolayer. The dependence of adsorption energy on electric field is presented in Fig. 7. As can be seen from Fig. 7, different gas molecules have differing response to the vertically applied electric field. For gas molecules that act as acceptor molecules (NO_2_, H_2_S, O_2_, N_2_), a positive electric field improved the value of adsorption energy (see Fig. 7(b, c, i and j)). In contrast, negative electric field improved the sensing response for other gas molecules. However, although the general trend for adsorption energy dependence on electric field was similar for CH_4_, CO_2_, and NO the adsorption energy under any electric field was greater than adsorption energy under zero electric field.

Since recovery time is directly dependent on the adsorption energy, a vertically applied electric field provided a relatively simpler reversible method for adsorption and desorption of gas molecules on the surface.

## 4 Conclusion

In conclusion, we have employed Density Functional Theory (DFT) calculations to investigate the adsorption properties and gas sensing potential of BeS monolayer. Our results reveal valuable insights into the electronic structure, optical properties, and charge distribution of BeS-analyte systems. Notably, we observe defect states in the band structure of BeS-analyte systems near the Fermi level, originating from the interaction between pollutant gas molecules and the BeS monolayer. The density of states analysis demonstrates a strong hybridization between the gas molecule orbitals and the BeS monolayer. Remarkably, the calculated adsorption energies for NH_3_ and CO adsorption on BeS monolayer (−1.24 eV and −0.51 eV, respectively) surpass those of widely studied gas sensing materials such as stanene, SiC, WSe_2_, and InN. Moreover, the charge transfer from CO or NH_3_ to the BeS monolayer is found to be higher compared to other 2D materials, with values of 0.227 |*e*| and 0.268 |*e*|, respectively.

Furthermore, we explore the optical properties of CO_2_ adsorbed on the BeS monolayer in the 100 to 200 nm range, revealing an 11% higher reflectivity and 10% higher absorption coefficient. The recovery time for gas desorption is rapid, on the order of nanoseconds, for all the gas molecules considered in our study, except for NH_3_. Notably, we find that tensile strain has minimal impact on the adsorption energy, except for NO, where tensile strain exceeding 2% facilitates desorption from the substrate. On the other hand, biaxial compressive strains significantly enhance the adsorption energy, thus enhancing the gas sensing performance of BeS monolayer.

Additionally, we investigate the influence of an external electric field on the adsorption energy. Our findings demonstrate that the adsorption energy can be finely tuned by applying a vertical electric field. This control over adsorption energy directly affects the recovery time, enabling precise manipulation of gas adsorption and desorption in the BeS monolayer by means of an electric field, such as a gate voltage in a 2D material transistor. Based on our comprehensive analysis, we propose that the high adsorption energy, low recovery time, and distinct optical properties of BeS monolayer position it as a promising candidate for future gas molecule sensing applications.

## Data availability

The datasets and computer codes are available upon request from the authors.

## Author contributions

Akib Zaman: conceptualization, methodology, software, validation, data curation, formal analysis, writing – original draft, writing – review & editing. Rifat Shahriar: conceptualization, methodology, software, validation, data curation, formal analysis, writing – original draft, writing – review & editing. S. M. Takvir Hossain: conceptualization, methodology, software, validation, data curation, formal analysis, writing – original draft, writing – review & editing. Md Rajbanul Akhond: formal analysis, writing – review & editing Homayra Tabassum Mumu: conceptualization, methodology, software, validation, data curation. Ahmed Sharif: conceptualization, methodology, writing – review & editing, resources, supervision.

## Conflicts of interest

The authors declare that they have no known competing financial interests or personal relationships that could have appeared to influence the work reported in this paper.

## Supplementary Material
